# Intraarticular injection of bone marrow-derived mesenchymal stem cells enhances regeneration in knee osteoarthritis

**DOI:** 10.1007/s00167-020-05859-z

**Published:** 2020-01-31

**Authors:** Emily Claire Doyle, Nicholas Martin Wragg, Samantha Louise Wilson

**Affiliations:** 1 National Centre for Sport and Exercise Medicine, School of Sport, Exercise and Health Sciences, Loughborough University, Epinal Way, Loughborough, LE11 3TU Leicestershire UK; 2 Centre for Biological Engineering, Wolfson School of Mechanical, Electrical and Manufacturing Engineering, Loughborough University, Epinal Way, Loughborough, LE11 3TU Leicestershire UK

**Keywords:** Allogenic, Autologous, Cell therapies, Clinical efficacy, Immunomodulation, Mesenchymal stem cells, Optimal dosage, Osteoarthritis

## Abstract

**Purpose:**

This review aimed to evaluate the efficacy of intra-articular injections of bone marrow derived mesenchymal stem cells (BM-MSCs) for the treatment of knee osteoarthritis (KOA).

**Methods:**

This narrative review evaluates recent English language clinical data and published research articles between 2014 and 2019. Key word search strings of (((“bone marrow-derived mesenchymal stem cell” OR “bone marrow mesenchymal stromal cell” OR “bone marrow stromal cell”)) AND (“osteoarthritis” OR “knee osteoarthritis”)) AND (“human” OR “clinical”))) AND “intra-articular injection” were used to identify relevant articles using PMC, Cochrane Library, Web Of Science and Scopus databases.

**Results:**

Pre-clinical studies have demonstrated successful, safe and encouraging results for articular cartilage repair and regeneration. This is concluded to be due to the multilineage differential potential, immunosuppressive and self-renewal capabilities of BM-MSCs, which have shown to augment pain and improve functional outcomes. Subsequently, clinical applications of intra-articular injections of BM-MSCs are steadily increasing, with most studies demonstrating a decrease in poor cartilage index, improvements in pain, function and Quality of Life (QoL); with moderate-to-high level evidence regarding safety for therapeutic administration. However, low confidence in clinical efficacy remains due to a plethora of heterogenous methodologies utilised, resulting in challenging study comparisons. A moderate number of cells (40 × 10^6^) were identified as most likely to achieve optimal responses in individuals with grade ≥ 2 KOA. Likewise, significant improvements were reported when using lower (24 × 10^6^) and higher (100 × 10^6^) cell numbers, although adverse effects including persistent pain and swelling were a consequence.

**Conclusion:**

Overall, the benefits of intra-articular injections of BM-MSCs were deemed to outweigh the adverse effects; thus, this treatment be considered as a future therapy strategy. To realise this, long-term large-scale randomised clinical trials are required to enable improved interpretations, to determine the validity of efficacy in future studies.

**Level of evidence:**

IV.

## Introduction

Osteoarthritis (OA) is one of the most ubiquitous joint disorders [1]; the prevalence of symptomatic hip and/or knee OA is ~ 242 million worldwide [2] with conditions ranked as the 11th highest contributors to global disability [1]. Decreased patient quality of life (QoL) and productivity poses a significant individual and societal burden, with a global prevalence of 3.8% [3, 4]. Knee OA (KOA) demonstrates higher incidences compared to other joints, with a lifetime risk of ~ 45%, increasing to 60.5% amongst obese patients [5]. Prevalence increases with each decade of life, with annual incidences highest between the ages of 55–65 years [6–9] further exacerbated by endogenous and exogenous risk factors (Table 1).

**Table 1:** Endogenous and exogenous risk factors for KOA

Endogenous	Exogenous
Age	Previous joint injuries
Incidence rates increase linearly in the 50–80 age range	
Sex	Body mass
Females have been reported to have a greater incidence rate compared to males	Overweight and obese people are significantly associated with higher KOA risk The risk increases by 35% with every 5 kg/m^2^ increase in BMI
Heredity	Excessive joint stress and increased mechanical forces
Genetics	Repetitive loading (kneeling and squatting)
Joint laxity	Occupation
	Physical work activities (kneeling/squatting/lifting and climbing) contribute to the occurrence/progression of KOA
Ethnic origin	Resective joint surgery
More common in individuals of European descent	
Post-menopausal changes	Muscle weakness
Malalignment	Lifestyle factors (alcohol, tobacco use)

Adapted from: Adatia et al. [64] and supplemented by Palmer [65]

OA results from degradation of the osteochondral unit composed of: articular cartilage; calcified cartilage; subchondral and trabecular bone, which synergistically support functional loading [10]. Throughout OA progression, degradative enzymes are overexpressed, including matrix metalloproteinases (MMPs) [11], which degrade both matrix and non-matrix proteins. Chondrocyte senescence and reduced cartilage elasticity [12] alters the tissue microenvironment impairing regeneration. Morphological changes in the subchondral bone include cartilage surface fibrillation and synovial fluid thickening [13, 14], accompanied by progressive synovitis and osteophyte formation. Progression according to imaging can be graded as localised (focal) or diffuse (affecting ≥ 75% of the region), and normal, doubtful, mild, moderate or severe (grade 0–4 on the Kellegren Lawrence scale) [15].

**Fig. 1:**
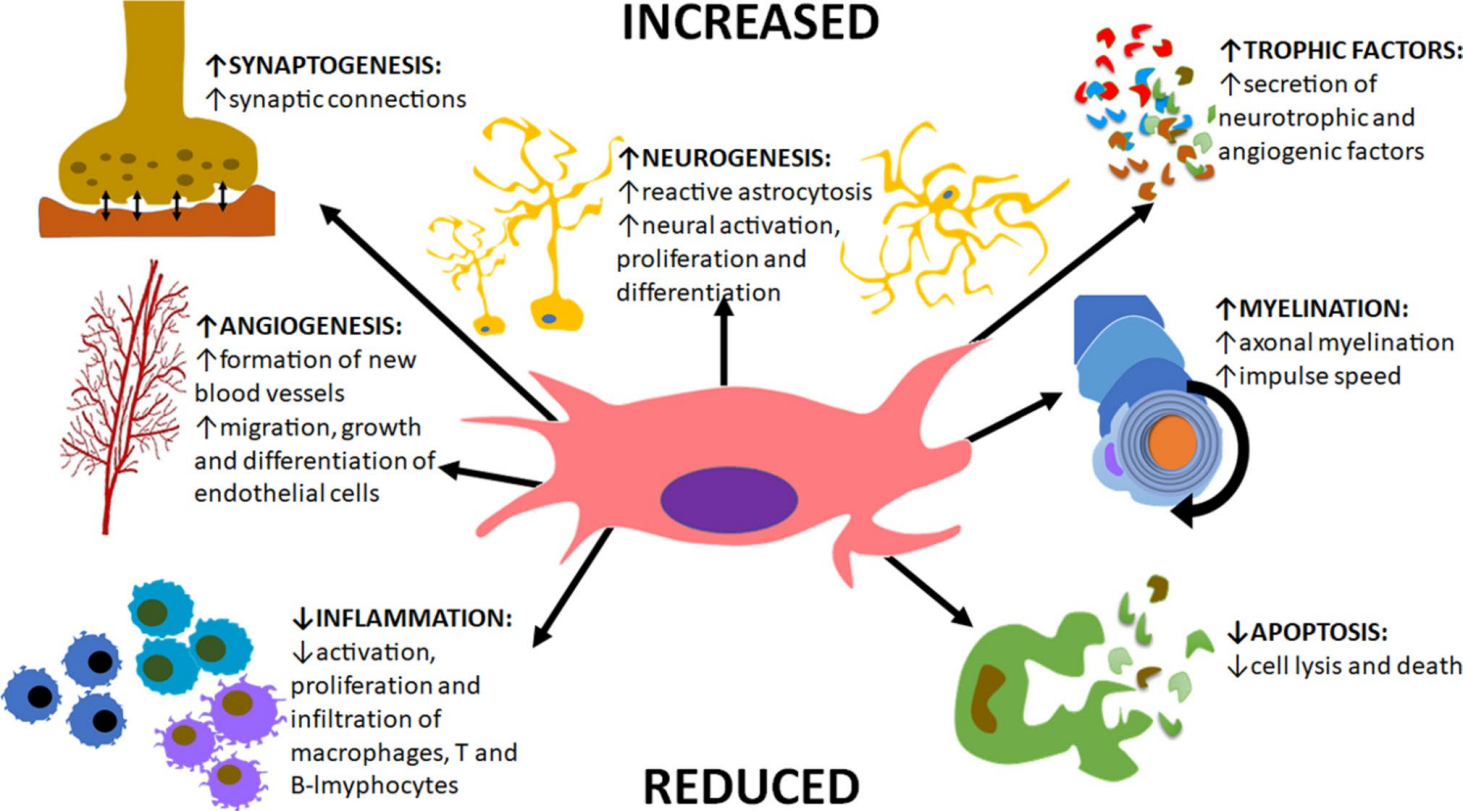
The function of MSCs play a significant role in the repair and regeneration process and are identified in this diagram. These include the reduction of cell death to continually replace lost cells, the secretion of trophic factors which stabilise the extracellular matrix and the suppression of immune cell activation to prevent inflammation. Adapted from: BioExplorer.net [63]

Currently, no conventional or pharmacological therapies have demonstrated unequivocal efficacy in halting disease progression and injections of molecular compounds to assist healing, such as corticosteroids, may only have placebic pain reducing effects [16–18]. Surgical interventions may be beneficial when mechanical deformity is present; however, therapeutic benefit is limited to late-stage OA and is not considered as a long-term solution. Alternatively, cellular regenerative therapies, including mesenchymal stem cells (MSCs) [19] and cell-derived products (such as platelet-rich plasma [20]) have shown therapeutic promise. Since OA is degenerative, likely involving endogenous MSC depletion, investigation into this therapy is supported by BM-MSCs inherent characteristics (Fig. 1) and their potential for articular cartilage repair/regeneration [21].

The purpose of this study was to review published literature to assess and evaluate the clinical efficacy of intra-articular injections of bone marrow-derived MSCs (BM-MSCs) specifically for KOA. It was hypothesized that BM-MSCs would have a beneficial impact on KOA clinical outcomes. This is clinically relevant since OA poses a significant individual, societal and economic global burden.

## Methods

A systematic literature search was performed using electronic databases PMC (PubMed), Cochrane Library, Web of Science and Scopus, to identify recent English language clinical data published between 2014 and 2019. Keyword search strings of (((“bone marrow-derived mesenchymal stem cell” OR “bone marrow mesenchymal stromal cell” OR “bone marrow stromal cell”)) AND (“osteoarthritis” OR “knee osteoarthritis”)) AND (“human” OR “clinical”))) AND “intra-articular injection” were applied. Cell dosages were analysed and compared. Despite results being predominantly positive, to increase confidence in clinical efficacy and comparability there needs to be a standardisation of methodologies including follow-up durations and appropriate controls and the application of quantitative outcome measures.

## Results

**Fig. 2:**
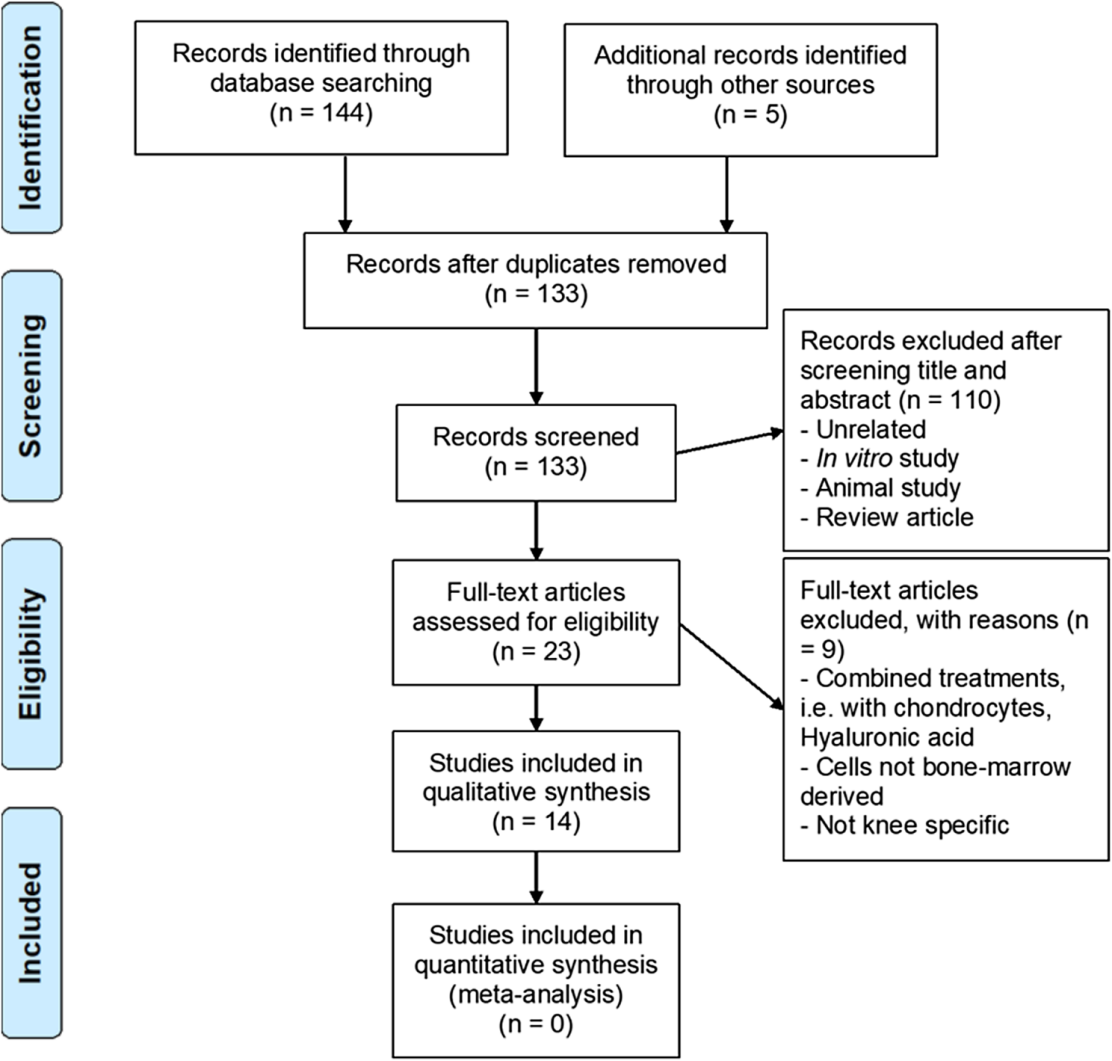
PRISMA flowchart of study selection criteria

Using the search terms described returned 139 records; 117 from PMS, 6 from Cochrane Library, 7 from Web of Science and 14 from Scopus respectively. A further five records were identified via other searches. The removal of duplicate records resulted in 133 records being screened for relevance (Fig. 2). The titles and abstracts were screened, and 100 records were removed since they were unrelated, in vitro studies, non-human studies and/or review articles. Of the 23 full-text articles assessed for eligibility, 9 records were excluded due to the methods employing combined treatments, i.e. BM-MSCs administered with chondrocytes or hyaluronic acid, the cells not being bone-marrow derived or the studies being non-knee specific. 14 studies were included in a qualitative synthesis. Due to the low number of published studies, coupled with the diversity of protocols implemented and significant variation in outcome measures applied it was extremely difficult to directly quantitatively compare studies. Thus, a narrative review was felt to be most appropriate to review and present the relevant literature.

## Discussion

### BM-MSCs for stimulating regeneration in knee osteoarthritis

Bone marrow tissue supports the complex microenvironment for numerous cell types and bone marrow aspirate can be used whole, concentrated, or as a source for stem cells [19]. BM-MSCs can be isolated from aspirate and have gained significant attention in the regenerative medicine field [22] due to their multilineage differentiation potential, immunomodulatory and self-renewal capacities [21, 23].

BM-MSCs promote repair via paracrine signalling mechanisms and the secretion of soluble trophic factors including bone morphogenetic protein-2 (BMP2) and insulin-like growth factor-1 (IGF1) [24]. These factors enhance cellular regeneration and induce bone formation by stimulating proliferation and differentiation of endogenous semi-like progenitors found in most tissues and by decreasing OA inflammatory and immune reactions [25]. BM-MSCs also inhibit T- and B-lymphocyte activation by inhibiting inflammatory cytokine production, thereby preventing immune responses and consequently promoting immune tolerance. Furthermore, BM-MSCs stimulate anti-inflammatory interleukin-1 (IL-1) supporting the generation of anti-inflammatory T-cells [26]. To guarantee these characteristics and standardise MSC classification, the Mesenchymal and Tissue Stem Cell Committee of the International Society for Cellular Therapy (ISCT) has defined MSC criteria (Table 2) to improve the validity and consistency of research trials.

**Table 2:** Committee of the ISCT criteria for the classification of mesenchymal stem cells [66]

Classification of MSCs
Fibroblastic-like (spindle-shaped) morphology
Plastic-adherent property under standard culture conditions
Differentiation potential into osteoblasts, adipocytes, and chondroblasts in vitro
Expression of surface markers including CD105; CD73 and; CD90
Lack of expression of: CD45; CD34; CD14; or CD11b; CD79α or CD19 and; HLA-DR

Pre-clinical studies investigating BM-MSCs for cartilage repair in animal models have demonstrated encouraging results (Table 3) [27–33]; subsequently, clinical applications are increasing (Table 4) [30, 34–44]. BM-MSCs administered for KOA in clinical patients adhere to damaged tissue surfaces, and differentiate into chondrocytes, resulting in anatomic restoration with significant improvements regarding pain and function [40, 43]. However, some studies have challenged whether BM-MSCs treatments are applicable to all OA grades [45–47]. Across research studies, a variety of outcome measures have been utilised, with some studies reliant upon qualitative questionnaires including The Western Ontario and McMaster Universities Osteoarthritis Index (WOMAC) and Lequesne algofunctional indexes [20] to evaluate success, which may introduce unintended bias [38, 42], due to physicians influencing patient responses. This may be improved by digital administration of questionnaires therefore, it will be completed individually with no external input [48].

**Table 3:** Overview of successful and fundamental pre-clinical studies that outline the safety and efficacy of BM-MSCs intra-articular injections (organised by animal model)

References	Animal model	Cell donor	Sample size	Severity of knee OA	BM-MSC dosage	Outcomes
Al Faqeh et al. [29]	Sheep	Autologous (chondrogenic-induced)	*n* = 51	Surgically induced (monitored for 24 h) 3-weeks post: Sheep ran 100 m on hard surface daily for 3 weeks	1 Injection: 2 × 10^6^ suspended in 10% foetal bovine serum	Meniscal regeneration and retardation of the progression of OA
Diekman et al. [28]	Mouse	Autologous (purified)	*n* = 11	Surgically induced: closed tibial plateau fracture	1 Injection: Experimental group: 1 × 10^5^ cells in 6 µl saline Control group: sterile saline solution	Efficacy in preventing OA
Murphy et al. [32]	Goat	Autologous	*n* = 24	Surgically induced: 3-weeks post: Sheep ran on 90 m hard surface daily for 3 weeks	1 Injection: Experimental group: 10 × 10^6^ suspended in 10% foetal bovine serum Control group: injection of hyaluronan acid (HA)	Meniscal regeneration and retardation of progressive destruction
Lee et al. [31]	Pig	Autologous	*n* = 27	Surgically induced	1 Injection: Group 1: MSCs with HA Group 2: HA Group 3: Saline solution	Cartilage repair
Gupta et al. [30]	Rat	Xenogeneic (human)	*n* = 74	Monoiodoacetate (MIA)-induced model of OA	1 Injection: Group 1: sham control received 60 μl of Plasmalyte A Group 2: vehicle control received 60 μl of Plasmalyte A Group 3: 30 μl of HA and 30 μl of vehicle 2 Injections: Group 4: 6 × 10^5^ of Stempeucel^®^ and 30 μl of HA Group 5: 1.3 × 10^6^ of Stempeucel^®^ and 30 μl of HA	Elicited pain reduction and cartilage regeneration
Suhaeb et al. [33]	Rat	Allogenic	*n* = 36	MIA injection	1 Injection: Control: No treatment Experimental group 1: 25 μl of HA Experimental group 2: 3–5 × 10^6^ cells Experimental group 3: 3–5 × 10^6^ cells + 25 μl of HA	Effective reduction of OA progression alone, compared with combined use of HA and BM-MSCs
Chiang et al. [27]	Rabbit	Allogenic	*n* = 2	ACLT	1 Injection: Group 1: OA induction without treatment Group 2: Sham operation Group 3: 0.4 mL of HA Group 4: 1 × 10^6^ and 0.4 mL of HA	Reduced OA progression

**Table 4:** Comparison of the most recent and pivotal clinical studies (ordered by study type/phase of trial and chronological date order)

References	Cell donor	Sample size/control	OA severity	BM-MSC dosage	Follow-up	Significant findings
*Case reports*						
Centeno et al. [35]	Autologous	*n* = 1 Male 56 years No control	OA causing significant on-going pain and disability (ungraded)	1 Injection: 22.4 × 10^6^ cells suspended in PBS Dexamethasone injection administered following BM-MSC injection	Baseline, 1 and 3 months	No adverse events reported MRI: ↑ cartilage and meniscus growth
Emadedin et al. [38]	Autologous	*n* = 6 Female Mean age: 54 years No control	Grade 4	1 Injection: 20–24 × 10^6^ cells suspended at a density of 5 × 10^6^	Baseline, 6 and 12 months	No local or systemic adverse events WOMAC: ↓ at 6 and 12 months Mean walking distance: ↑ at 6 months No local or systemic adverse events Mean walking distance: ↑ at 6 and 30 months after treatment WOMAC: ↓ at 6, 12 and 30 months compared with baseline. ↓ in WOMAC physical function sub scores at 6 and 12 months
Emadedin et al. [39] Long term-follow up	Autologous	*n* = 6 Female Mean age: 54 years No control	Grade 4	1 Injection: 20–24 × 10^6^ cells suspended at a density of 5 × 10^6^	Baseline, 6, 12 and 30 months	
Mehrabani et al. [67]	Autologous	*n* = 1 Female 47 years No control	Grade 4 Unresponsive to NSAIDs	1 Injection: 36 × 10^6^ cells provided and transferred in 2 ml of media	3, 6 and 12 months	No local or systemic adverse events MRI: ↑ thickness of cartilage on distal condyle of femur and proximal tibia at 6 and 12 months
*Preliminary reports*						
Davatchi et al. [36]	Autologous	*n* = 4 2 Females: 57 and 54 years 2 Males: 55 and 65 years	Grade 2–3	1 Injection: 8–9 × 10^6^ in a mean volume of 5.5 mL	Baseline, 1 week, then every month up to 1 year	No adverse events reported
Davatchi et al. [37] 5-year follow-up	Autologous	*n* = 3 No control	Grade 2–3	1 Injection: 8–9 × 10^6^ in a mean volume of 5.5 mL	5 years	No adverse events reported
*Pilot studies*						
Orozco et al. [40]	Autologous (according to Good Manufacturing Processes: GMP)	*n* = 12 6 Females 6 Males Mean age: 49 ± 5 No control	Grade 2 to 4 Unresponsive to conservative treatment for 6 months	1 Injection: 40 × 10^6^ cells suspended in ringer-lactate at 5 × 10^6^	Baseline, 3, 6 and 12 months	Mild adverse events: the first 1–6 days and occurred frequently (50% of patients) = controlled by ibuprofen VAS: Pain ↓ at 3 months with progressive improvement during the subsequent 9 months (statistically significant at all time points compared with basal pain level) WOMAC: All subscales ↓ at 12 months compared to baseline Lequesne algofunctional index: Correlation between improvement and the initial score (*p* < 0.01) MRI: Mean PCI ↓ from 19.5 to 15.4 during first 6 months and further ↓ to 14.3 at 12 months (11 out of 12 patients) Correlation between VAS and PCI
Orozco et al. [40] Two-year follow up	Autologous (GMP)	*n* = 12 6 Females 6 Males Mean age: 49 ± 5 No control	Grade 2 to 4 Unresponsive to conservative treatment for 6 months	1 Injection: 40 × 10^6^ cells suspended in ringer-lactate at 5 × 10^6^	2 years	Results of the follow-up reaffirm the conclusions for the first-year results regarding feasibility and safety
*Phase I/II studies*						
Rich et al. [41] Clinical trial-Phase I/II	Autologous (*ex-vivo*)	*n* = 50 20 Females 30 Males Mean age: 57.8 ± 14.1 No control	Grade 2–4	1 Injection: 40 × 10^6^ suspended in ringer-lactate at 5 × 10^6^	Day 8, 3, 6 and 12 months	No local or systemic adverse events VAS: ↓ at 6 and 12 months The pattern of 1-year improvement was parallel for VAS, WOMAC and Lequesne algofunctional index MRI: Mean PCI ↓ from 25 to 5 at 12 months post-injection
Soler et al. [42] Prospective, open-label, single-dose, single-arm clinical trial-Phase I/II final results	Autologous (*ex-*vivo)-Infusion of XCEL-M-Alpha	*n* = 15 9 Females 6 Males Mean age: 52 No control	Grade 2 (*n* = 9) Grade 3 (*n* = 6)	1 Injection: 40 × 10^6^ ± 10 × 10^6^ XCEL-M-ALPHA was infused within 6 h from delivery	Day 8, 3, 6- and 12-months	Mild adverse events (local discomfort and back pain from bone marrow extraction) VAS: Pain ↓ and daily activity ↑ at 8 days until the end of the study period HAQ (questionnaire): Over time ↓ from baseline across whole population at 12 months WOMAC: ↓ at 12 months Lequesne algofunctional index: ↓ at 6 and 12 months
Al Najar et al. [34] Prospective open-label study-Phase I/II	Autologous	*n* = 13 7 Females 6 Males Mean age: 50 years No control	Grade 2–4	2 Injections (1 month apart): 30.5 × 10^6^ cells suspended in 09% normal saline	Adverse events: day 1, 7, 14, 28, 60 and then every 6 months until month 24 Normalised KOOS: baseline, 1, 2, 4, 6, 12 and 24 months after first injection MRI: baseline, 6 and 12 months	2 local adverse events within 2 h of injection, 1 6 h after injection (all resolved with ice/mild analgesia in 48 h) Normalised KOOS: Symptoms and pain ↓, daily life activity, sport and QoL ↑ at 6, 12 and 24 months MRI: ↑ Mean tibial and femoral plate thickness (mm) at 12 months (1 female deteriorated by MRI despite of KOOS improvement)
*Randomised clinical trails (RCT)*						
Vangsness et al. [43] Double-blinded, randomised, controlled clinical study	Allogenic (GMP) Obtained from donors (screened and tested according to the US FDA)	*n* = 55 63% were male Group A = 18 Group B = 18 Control = 19 Mean age: 46 years	All underwent subtotal meniscectomies-any previous knee ligament reconstruction needed to have had a stable result	Group A: 50 × 10^6^ cells suspended in 2 mL of HA, human serum albumin and PlasmaLyte A to a volume of 5 ml Group B: 150 × 10^6^ suspended in 2 mL of HA, human serum albumin and PlasmaLyte A to a volume of 5 ml Control: A vehicle control compromised the same HA solution without BM-MSCs	Baseline, 6 weeks, 6 months, 1 year and 2 years post-operatively	427 adverse events among 55 patients. 272 were mild, 28 sever and 1 life-threatening 1-year post VAS: Knee pain ↓ for all patient’s compared with baselines in all groups. Significant differences were observed at 2 years for group A, and at 1 and 2 years for B Lysholm knee scale: Total scores relative to baseline ↓ at all follow-ups Meniscus volume > 15%: At 12 months, both the control compared with group A and overall comparison were significant (> 15%) and at 2 years the overall group comparison was significant
Vega et al. [44] Randomised controlled, comparator multi-centre-Phase I/II study	Allogenic (GMP)	*n* = 30 17 Females 13 Males Mean age: 57 ± 9 Experimental group = 15 Control = 15	Grade 2–4 Unresponsive to conventional treatments for at least 6 months prior to recruitment	1 Injection: Experimental group: 40 × 10^6^ cells from a 5 × 10^6^ cell/mL suspension Control: 60 mg of HA in 3 mL	Baseline, day 8, 3, 6 and 12 months	Minor adverse events during first 7 days in both groups = 53–60% of patients AS: ↓ in experimental group at 6 and 12 months. Control group ↓ at 12 months WOMAC: Pain and general WOMAC ↓ at 6 and 12 months for experimental group Lequesne algofunctional index: ↓ at 6 and 12 months in experimental group MRI: Poor Cartilage Index (PCI) ↓ at 12 months in experimental group
Vangsness et al. [43] Double-blinded, randomised, controlled clinical study	Allogenic (GMP) (screened and tested according to the US FDA)	*n* = 55 63% were male Group A = 18 Group B = 18 Control = 19 Mean age: 46 years	All underwent subtotal meniscectomies-any previous knee ligament reconstruction needed to have had a stable result	Group A: 50 × 10^6^ cells suspended in 2 mL of HA, human serum albumin and PlasmaLyte A to a volume of 5 ml Group B: 150 × 10^6^ suspended in 2 mL of HA, human serum albumin and PlasmaLyte A to a volume of 5 ml Control: A vehicle control compromised the same HA solution without BM-MSCs	Baseline, 6 weeks, 6 months, 1 year and 2 years postoperatively	427 adverse events among 55 patients. 272 were mild, 28 sever and 1 life-threatening 1-year post VAS: Knee pain ↓ for all patient’s compared with baselines in all groups. Significant differences were observed at 2 years for group A, and at 1 and 2 years for B Lysholm knee scale: Total scores relative to baseline ↓ at all follow-ups Meniscus volume > 15%: At 12 months, both the control compared with group A and overall comparison were significant (> 15%) and at 2 years the overall group comparison was significant
Vega et al. [44] Randomised controlled, comparator multi-centre- Phase I/II study	Allogenic (GMP) Obtained from three healthy donors	*n* = 30 17 Females 13 Males Mean age: 57 ± 9 Experimental group = 15 Control = 15	Grade 2–4 Unresponsive to conventional treatments for at least 6 months prior to recruitment	1 Injection: Experimental group: 40 × 10^6^ cells from a 5 × 10^6^ cell/mL suspension Control: 60 mg of HA in 3 mL	Baseline, day 8, 3, 6 and 12 months	Minor adverse events during first 7 days in both groups = 53–60% of patients VAS: ↓ in experimental group at 6 and 12 months. Control group ↓ at 12 months WOMAC: Pain and general WOMAC ↓ at 6 and 12 months for experimental group Lequesne algofunctional index: ↓ at 6 and 12 months in experimental group MRI: Poor Cartilage Index (PCI) ↓ at 12 months in experimental group
Gupta et al. [30] Randomised, double-blind multicentre placebo-controlled phase II study	Allogenic (GMP *ex-vivo*) Stempeucel^®^	*n* = 60 15 in each dose group (4 groups) Randomised into two groups within the 4 main groups (2:1) for Stempeucel^®^ and placebo (control)	Grade 2–3	1 Injection: Group 1: 25 × 10^6^ of Stempeucel^®^ Group 2: 50 × 10^6^ of Stempeucel^®^ Group 3: 75 × 10^6^ of Stempeucel^®^ Group 4: 150 × 10^6^ of Stempeucel^®^ Each group control: PlasmaLyte placebo	Baseline, 1 week, 1, 3 and 6 months Clinical data unblinded after 6 months but followed-up until 12 months	Adverse events: 97 mild to moderate adverse events were reported in 40 subjects
Espinosa et al. [49] Randomised clinical trial Phase I/II	Autologous (GMP) co-administered with HA	*n* = 30 Control group:10 Low dose:10 High dose:10 Active control	Inclusion of ≥ 2 Range: 2–4	1 Injection: Control: 60 mg HA (in a volume of 4 ml) Low BM-MSC dose: 10 × 10^6^ cells in 1.5 ml ringer’s lactate solution + 4 ml of HA injection High BM-MSCs dose: 100 × 10^6^ cells in 3 ml ringer’s lactate solution + 4 ml of HA injection	Baseline, 3, 6 and 12 months	Articular pain requiring anti-inflammatory treatment at 24 h post-injection in 1, 3 and 6 patients in the control, low-dose and high-dose groups respectively Range of motion: ↑ in BM-MSCs treated groups (effect seen earlier in higher dose) VAS score ↓ in low and high dosage groups at all follow-up times WOMAC: Control: Pain ↓ at 3 and 6 months, and function ↑ at 3 and 6 months Low dose: Stiffness ↓ at 6 and 12 months High dose: All WOMAC sub-scores improved significantly at 12 months X-ray: Knee joint space ↓ in control group at 12 months

Within the literature reviewed, the follow-up periods and outcome criteria varied. Periods up to 12-month post-injection have been followed, with clinical outcomes including increased cartilage thickness, function and pain measured [34]. Despite reported improvements, not all are significant. Contrastingly, greater BM-MSC longevity with significant changes in both qualitative and quantitative after-effects have been reported [34, 44, 49]. An injection of 40 × 10^6^ cells in 12 patients with advanced KOA displayed significant improvements in VAS and quality of articular cartilage without diminution between a 12 [40] and 24-month follow-up [44]. Davatchi et al*.* [37] reported a 5-year follow-up post injection of 8–9 × 10^6^ BM-MSCs in 4 patients and observed progressive deterioration, although outcomes were improved compared to baseline measurements, suggesting a protective role of BM-MSCs compared to untreated controls. Despite prolonged follow-up periods, limited patient numbers and lack of in-depth statistical analysis make it difficult to draw robust conclusions regarding the overall therapeutic efficacy [50].

Compared to autologous BM-MSCs, allogenic BM-MSCs represent an alternative cell source. Multiple randomised control trials (RCTs) have reported improved outcomes with various doses (25 × 10^6^–50 × 10^6^ cells), which are safe and well tolerated, whereas higher doses can produce adverse events [30]. Despite positive trends in similar studies [43, 44], few clinical parameters were significantly improved; with no critical changes in X-ray and Magnetic Resonance Imaging (MRI) compared to baseline measurements. Although BM-MSCs are considered poorly immunogenic, allogenic cells may stimulate immune responses and thus, the identification of an optimum dose is crucial for viable treatment strategies [50].

There is currently limited evidence for simultaneous improved clinical outcomes, including pain, function, and cartilage repair. However, improvements following the application of intra-articular BM-MSCs at short-term follow-up have been reported [22]. Several studies have reported improved cartilage thickness; however, meaningful changes in clinical outcomes are sporadic. Moreover, studies using both autologous and allogenic BM-MSCs have been explored within the literature, including co-administered *and ex-vivo* expanded treatments [30, 49].

### Critique of BM-MSC intra-articular injections for the treatment of knee osteoarthritis

Autologous BM-MSC injections are the dominant cell choice in clinical studies reported for treating KOA [51]. In an early case report [35], a single patient was injected with 22.4 × 10^6^ cells suspended in phosphate-buffered saline (PBS) with a dexamethasone post-injection as a differentiating agent [52]. The 3-month follow-up reported no adverse events, with significantly increased cartilage and meniscus growth, with minor improvements in range of motion (ROM) and pain scores. This was the first report of increased meniscus size in humans; however, the methodology lacked specificity and a detailed exploration of the dexamethasone effects was not conveyed [52]. MRI revealed significant cartilage thickening covering the distal femur and proximal tibia at 6 and 12 months, yet symptomatic and functional improvements were not apparent. This study failed to acknowledge potential author or methodological bias and so further pilot and clinical studies are required to replicate meaningful findings.

Davatchi et al*.* [36] emphasised the safety of BM-MSC injections, claiming marked improvements in (qualitative) outcome parameters, with physical parameters improving to a lesser extent. In comparison to Centeno et al*.* [35], a lower dosage of 8–9 × 10^6^ BM-MSCs were administered, potentially accounting for lower physical parameter improvements. The follow-up from a 2011 case series [37] argued that the lack of significant outcomes is due to all participants having advanced-stage OA.

Emadedin et al*.* [38] performed a similar study on a small patient cohort (*n* = 6), using 20–24 × 10^6^ cells, reporting significant improvements in pain and function (WOMAC) at both 6 and 12 months. A long-term follow-up of the same cohort affirmed previous findings, revealing that BM-MSC dosages were safe and therapeutically beneficial. Nevertheless, between 12 and 30 months, therapeutic improvements declined in all individuals, suggesting the need for subsequent administration for prolonged benefit [39].

Despite reported therapeutic benefits of BM-MSCs, the generalisability of the results and techniques used for larger populations with symptomatic KOA is limited. This highlights the requirement for larger, blinded RCTs to improve study comparability and clinical validity. Likewise, within study designs, sample size calculations should be utilised for methodological and ethical reasons. Otherwise, reported findings should be interpreted with caution, as smaller samples may undermine internal and external study validity.

A clinical RCT (phase I/II) of 30 patients with grade ≥ 2 OA used a sample size calculation that provided an effect size of 0.6 and, a power of 80% [49]. Group randomisation was performed, potentially facilitating the increased cohort size compared to previous studies (*n* = 30), whilst reducing bias [53]. Despite randomisation, the stage of OA was more severe in those receiving low-dose BM-MSCs (10 × 10^6^), which may have prevented these patients achieving more positive outcomes [49]. The study included blinded radiologists to reduce bias (extra KOA MRIs were randomly added during analysis). Ethical issues prevented double-blinding; thus, qualitative clinical scores were compared with objective measures to minimise bias. A control, low-dose and high-dose group were followed for 12 months. Outcomes were significant at 3, 6 and 12 months and correlations revealed a significant reduction in low-and-high-dose VAS scores at all time points, which correlated with improved ROM compared to the control group. Importantly, this study co-administered hyaluronic acid (HA) and indicated that a single injection is a safe and feasible procedure, resulting in both clinical and functional improvements; particularly when 100 × 10^6^ cells were administered.

Additional studies using allogenic BM-MSCs also used HA either as a control group or as a cell suspension [43, 44]. However, cells from young, healthy donors may not reflect growth and differentiation characteristics of MSCs from elderly and/or OA patients [54]. Yet, they equally showed improvements in pain and function. Espinosa et al*.* [55] described a decrease in knee joint space in control groups (HA) at 12 months. Whereas Vangsness et al*.* and Vega et al*.* [43, 44] provided MRI analysis using T2 mapping of cartilage and, computational analysis of meniscus volume to assess the effects of BM-MSCs more closely. Vega et al*.* [44] found significant decreases in poor cartilage index (PCI) at 12-month following injection of 40 × 10^6^ cells. Conversely, a dosage of 50 × 10^6^ cells [43] identified significantly increased meniscus volume (> 15%) at 12 months, which continued 2 years post-injection. Orozco et al*.* [40] also reported significant improvements in PCI following treatment with autologous BM-MSCs with continued improvement over the 2-year follow-up. A significant correlation between VAS and PCI was reported, demonstrating that both cartilage and pain/function improvements occur simultaneously. This study supersedes previous case reports where results were described as “satisfactory” [39], as cell dosages were larger, the follow-up was longer and the MRI investigation provided robust quantitative analysis.

Alternative methodologies for the application of MSCs into KOA patients exist, however these are more invasive but do allow more specific targeting of focal cartilage defects. Brittberg et al. and Bornes et al. have both comprehensively discussed the use of MSCs (not just limited to BM-MSCs) in the specific treatment of cartilage defects considering multiple therapy options [56, 57].

### Safety considerations when using BM-MSCs for the treatment of KOA

The feasibility and safety of both allogenic and autologous cells have been reiterated throughout literature [40, 41]; yet, studies utilising higher doses regularly report increased adverse events. In a sample of 55 patients, 247 adverse events were reported, with one life-threatening, 1-year post-injection when using allogenic BM-MSCs [43]. Minor adverse events, such as post-implantation pain and inflammation, occur with similar frequencies (50%) between studies using autologous BM-MSCs [40, 49]. Most adverse events were resolved within 24–48 h following treatment with pain medication. Nevertheless, adverse events were not reported in every study [35, 39, 41].

Close attention to adverse events may be key to clinical translation when optimising BM-MSCs as a KOA therapy [51]. The majority of literature reports use of either autologous or allogenic BM-MSCs, however studies have also included infused BM-MSCS [30, 42]. Soler et al*.* [42] produced an analysis of a prospective, open-label, single-arm clinical trial for the infusion of XCEL-M-Alpha into autologous BM-MSCs. Significant improvements in VAS, WOMAC and Lequesne algofunctional index were observed following a 40 × 10^6^ injection. However, this is one of the first to infuse BM-MSCs and the omission of control groups makes it difficult to evaluate efficacy. The indistinct method regarding infusion is inconsistent with the detailed explanation of cell isolation and expansion and therefore, the methodology may be lacking suitable description. Furthermore, Gupta et al*.* [30] injected ex vivo expanded, pooled allogenic BM-MSCs (Stempeucel^®^) into 60 patients who were split into four different dosage groups (with a control each) in a randomised, double-blinded multicentre placebo-controlled study (RCT). However, unblinding of the trial occurred after 6-month follow-up even though subjective measurements were continuously analysed; after this point, results should be interpreted with circumspection. The therapeutic effect of BM-MSCs was not explored without HA, but both pre-clinical and clinical studies suggest that BM-MSCs co-administered with HA tends to produce greater regenerative benefit [58]. This study also failed to produce any significant outcomes, which was potentially due to the procedure employed with higher dosages and volumes (75 and 150 million cells) being restricted in the limited joint space; possibly causing cell aggregation. The study may have been more valuable if lower dosages were also examined.

Overall, there is moderate-to-high level evidence of safety to recommend therapeutic administration of BM-MSCs for KOA, for both animal and human studies; suggesting therapeutic benefit. Several published results, especially conclusions and speculations drawn from case/preliminary reports, do not have the weight of findings compared to RCTs. Therefore, when interpreting results, due diligence is recommended. Furthermore, the significance of experimental outcomes may be influenced by the prevalent study heterogeneity including: the use of a variety of cell doses and donors; variability in functionality and pain scores; severity of KOA; various cell processing methods and differing follow-up periods [59].

Due to the immune-privileged status of MSCs [60], allogenic BM-MSCs show more promise compared to autologous, since they allow manufacturing of large batches or ‘off-the-shelf’ products in the future [61]. This would enhance the reliability of production whilst decreasing the costs of cell therapies [62], however, long-term efficacy data are warranted.

Conflicting study results may result from methodological heterogeneity or, the limitation of BM-MSCs remaining localised within the tissue. This may be caused by the rapid cycling of synovial fluid or due to large volumes injected into the knee causing cell apoptosis. Despite reported adverse events, the outcomes across the published studies are influential in demonstrating that the benefits may outweigh the treatment risks. Despite BM-MSCs intra-articular injections potentially having a limited therapeutic effect on cartilage volume [51], the clinical and functional outcomes are favourable in patients with chronic KOA. In terms of evaluating BM-MSC efficacy, it may be more applicable for future studies to only focus on long-term, large-scale RCTs as non-RCTs tend to have greater bias and more confounders, affecting the interpretation and validity of efficacy [22]. Future studies need to determine the type and quality of the repaired cartilage tissues, its durability and the association between objective and subjective outcome improvements [22].

## Conclusion

It is apparent that moderate-high cell numbers (40 × 10^6^) are most likely to achieve optimal responses in individuals with grade ≥ 2 KOA. The highest number of cells used (100 × 10^6^) also produced significant improvements in KOA, although this came at a cost, with greater risks of adverse events. Therefore, the number and type of cell donor BM-MSCs, the timing of injection, the stage of the disease and the number of injections requires further investigation to achieve optimal therapeutic benefit.

Subsequently, a unified classification of intra-articular dosage and efficacy needs to be agreed before safe and effective treatment can be implemented as a leading regenerative treatment strategy, across all OA populations.

## Abbreviations

OA: Osteoarthritis
QoL: Quality of life
BM-MSCs: Bone marrow derived-mesenchymal stem cells
KOA: Knee osteoarthritis
WHO: World Health Organisation
MMPs: Matrix metalloproteinases
BMP2: Bone morphogenetic protein 2
IGF1: Insulin-like growth factor 1
IL-1: Interleukin-1
ISCT: International Society for Cellular Therapy
WOMAC: The Western Ontario and McMaster Universities Osteoarthritis Index
VAS: Visual analogue scale
KOOS: Knee injury and osteoarthritis outcome score
MRI: Magnetic resonance imaging
PBS: Phosphate-buffered saline
RCT: Randomised control trial
